# Effect of systemic antibiotic and probiotic therapies as adjuvant treatments of subgingival instrumentation for periodontitis: a randomized controlled clinical study *

**DOI:** 10.1590/1678-7757-2021-0583

**Published:** 2022-03-23

**Authors:** Tatiane Caroline de Souza Ramos, Mariéllen Longo Vilas Boas, Camilla Magnoni Moretto Nunes, Camila Lopes Ferreira, Cláudio Mendes Pannuti, Mauro Pedrine Santamaria, Maria Aparecida Neves Jardini

**Affiliations:** 1 Universidade Estadual Paulista Faculdade de Odontologia São José dos Campos SP Brasil Universidade Estadual Paulista (UNESP), Faculdade de Odontologia, Programa de Biopatologia Bucal, Área de Periodontia, São José dos Campos, SP, Brasil.; 2 Universidade de São Paulo Faculdade de Odontologia Divisão de Periodontia São Paulo SP Brasil Universidade de São Paulo (USP), Faculdade de Odontologia, Divisão de Periodontia, Departamento de Estomatologia, São Paulo, SP, Brasil.; 3 Universidade Estadual Paulista Instituto de Ciências e Tecnologia Departamento de Diagnóstico e Cirurgia São José dos Campos SP Brasil Universidade Estadual Paulista (UNESP), Instituto de Ciências e Tecnologia, Departamento de Diagnóstico e Cirurgia, Área de Periodontia, São José dos Campos, SP, Brasil.

**Keywords:** Periodontitis, Periodontal debridement, Antibiotics, Probiotics

## Abstract

**Objective:**

This study assessed the efficacy of two adjunct therapies (antibiotic and probiotic) for periodontal treatment based on clinical and immunological parameters in patients with Stage II and III Grade B periodontitis.

**Methodology:**

45 patients were randomly allocated into three groups: control group (CG); antibiotic group (GAtb), in which 500 mg amoxicillin + 400 mg metronidazole were used; and probiotic group (GProb), for which *Lactobacillus reuteri* was used. Patients received medications after undergoing periodontal debridement. Clinical and immunological parameters were assessed at baseline, 30 days, and 90 days.

**Results:**

All therapies reduced bleeding on probing (BoP) in the evaluated periods, and the GAtb had a greater reduction at 90 days (p=0.03). The GProb group showed better results for plaque index (PI) and gingival recession (GR) compared to the GAtb at 90 days (p=0.0014; p=0.006). The area of inflammation (PISA Index) significantly decreased in all therapies in the evaluated periods. Therapies had no significant differences regarding moderate pockets. The GAtb had a greater reduction in probing depth (PD) for deep pockets (p=0.03) at 90 days and in the number of deep pocket sites at 30 days (p=0.04). The occurrence of adverse effects was commonly reported in the GAtb as a percentage per patient. The GAtb had a significant reduction in the concentration of interleukins IL-1β and IL-8 and an increase in IL-10 and TNF-α. The CG had a reduction in IL-6 and IL-1 β, whereas in the GProb there was no difference.

**Conclusion:**

After three months, none of the adjuvant therapies provided any additional benefit for subgingival instrumentation.

## Introduction

Periodontitis is a multifactorial chronic inflammatory disease associated with biofilm dysbiosis and characterized by the progressive destruction of the dental support apparatus, which is initially treated nonsurgically.^1^

Antibiotics can be prescribed for patients who do not respond to conventional mechanical therapy or patients with acute periodontal infections associated with systemic manifestations – including prophylaxis, in patients with systemic involvement – and as an adjunct to surgical and nonsurgical periodontal therapy.^2 , 3^ Antibiotic therapy is expected to reduce or eliminate pathogenic microorganisms that were not accessed by mechanical removal.^2^ The literature^4 , 5^ shows that, together with nonsurgical periodontal therapy, systemic antibiotics can show positive results and clinical improvements, which are however accompanied by adverse effects, including gastrointestinal discomfort, nausea, oral ulcers, diarrhea, and burning sensation, among others.

Therefore, other adjuvant mechanisms are essential to fight the disease as alternatives to the use of antibiotics, avoiding the excessive use and occurrence of bacterial strains resistant to the antibiotics currently available. The literature has proposed the modulation of the biofilm composition by simultaneous probiotics administration and periodontal debridement.^6 – 10^

The reason for using probiotics as an adjunct to conventional periodontal therapy is based on the etiology of periodontal inflammation related to bacterial plaque. This etiological view considers three factors as determinants of the development of the disease in an individual: a susceptible host, the presence of pathogenic species, and the reduction or absence of so-called “beneficial bacteria.”^7^

Probiotic organisms can be used for a number of mechanisms,^11^ including: 1) exclusion and competition with possible pathogens for nutrients and cell and epithelial adhesion; 2) production of antibacterial substances against periodontopathogens; and 3) local and systemic immunomodulation by regulating the expression of phagocytosis receptors in neutrophils, increasing the activity of natural killer (NK) cells, and being recognized by host cells such as epithelial cells and immune cells, thus increasing the mucosal barrier function.^7 , 12^

To date, the most commonly used probiotic species for the treatment of patients with periodontitis is *Lactobacillus reuteri* . Using lozenges once or twice a day has been considered a useful adjuvant therapy for periodontal debridement. Lower plaque index (PI), gingival index (GI), probing depth (PD), and bleeding on probing (BoP) were observed after treatment with this probiotic species.^13^

Few randomized controlled clinical studies have assessed the effects of probiotics as adjuvant therapy in periodontal debridement, which are still inconclusive. This study aimed to assess, using clinical parameters, the response of Grade B and Stage II and III periodontitis to two adjuvant therapies (antibiotic and probiotic). Considering the benefits of probiotics for general health and for the immunological system, we hypothesize that adjuvant probiotics provide additional benefits for subgingival instrumentation regarding pocket depth reduction.

## Methodology

### Study design

The study's methodology followed the standards of Consort 2010 Statement and SPIRIT 2013 Statement. A randomized controlled clinical trial was registered in ClinicalTrials.gov (NCT69281903) and approved by the Human Research Ethics Committee of the Institute of Science and Technology at Universidade Estadual Paulista (UNESP) (protocol number 2,708,853). All patients provided their informed consent to participate in the study.

### Study Population

Participants were selected from February to October 2019 and sent to the Periodontics division of the Institute of Science and Technology of UNESP, Brazil. The following inclusion criteria were used: 1) individuals between 35 and 50 years old 2) who had at least 18 teeth,^14^ 3) at least six sites per patient (three sites with moderate pockets with BoP and three sites with deep pockets with BoP), 4) had good systemic health, 5) agreed to participate in the study and signed the Informed Consent Form. The exclusion criteria were: 1) patients with systemic problems (cardiovascular changes, diabetes, blood dyscrasias, and immunodeficiencies, among others) that contraindicated the periodontal procedure; 2) had underwent periodontal treatment in the last 12 months; 3) had used antibiotics, anti-inflammatories, or probiotic supplements in the last 6 months; 4) were smokers; 5) were pregnant or lactating; and 6) chronically used medications that could affect the periodontal response.

### Initial therapy

All patients received information regarding their periodontal condition and oral hygiene guidelines. Biofilm, supragingival calculus, teeth indicated for extraction, and excessive restorations were removed, and endodontic treatments and cavity filling were performed.

### Randomization and allocation concealment

A person external to the research generated a simple random computer sequence to allocate patients to each group. This sequence was kept in brown/opaque and sealed envelopes. Each envelope had a number regarding the patient's group after the debridement session. This measure aimed to hide the randomization sequence from the professional responsible for treatment and clinical measures.^15^

### Treatment

All patients received ultrasonic periodontal debridement of the full mouth in a single stage.^14^ They were anesthetized with a sterile injectable solution of 2% mepivacaine hydrochloride (20 mg/ml) associated with epinephrine 1:100.000 (0.01 mg/ml) (DLA Pharmaceutical Ltda. – Catanduva, SP, Brazil) and the session lasted an average of one hour. A ultrasonic equipment (ProfiNeo, Dabi Atlante – Brazil) with specific tips (Dabi Atlante Tip Perio Sub – EVMWQHED3 – Brazil) was used. All sites affected by periodontal disease were instrumented in this debridement session, conducted by a single trained operator (MANJ) blinded to the allocation of patients and not involved in the outcomes assessment. After this mechanical therapy, patients were assigned to the control (CG), debridement + antibiotic (GAtb), and debridement + probiotic (GProb) groups.

The probiotic used was *Lactobacillus reuteri* (DSM 17938 and ATCC PTA 5289) at a dose of 2 x 10^8^ CFU/tablet (200 million live *Lactobacillus reuteri* bacteria per tablet) (BioGaia Prodentis – Lund, Sweden). Administration started after the periodontal debridement session. Patients were instructed verbally and in writing to use the lozenges twice a day, after brushing their teeth in the morning and at night, for 21 consecutive days.^8 , 12^

The antibiotics used were 500 mg amoxicillin (Sanofi Medley^®^, Brazil) in capsules and 400 mg metronidazole (Flagyl^®^, Sanofi Medley^®^, Brazil) in coated tablets. Patients were instructed verbally and in writing to take the medications together every eight hours for seven days.^16^

The groups were instructed to look for any possible symptoms or side effects, including malaise, dizziness, drowsiness, diarrhea, itching, and skin rash, and write them on a form provided by the researchers.^17^

At the end of treatment, a 30- and 90-day follow-up was conducted.

### Clinical measures

All clinical measurements were performed by a single investigator (TCSR) previously calibrated and blinded to the treatment received by the patients. The examiner was calibrated by measuring the probing depth and the clinical attachment level (CAL) of ten patients twice in a 48-hour interval. Measurements were then submitted to the intraclass correlation test, and the examiner was judged and calibrated if a 90% agreement of the measurements was achieved. The procedure was repeated until the examiner reached this index.

Assessments were performed before treatment (baseline) and 30 and 90 days after treatment. The following parameters were evaluated: 1) PI^18^ ; 2) BoP; 3) PD, with a North Carolina periodontal probe (UNC – Hu-Friedy); and 5) gingival recession (GR). The periodontal inflamed surface area (PISA) was calculated according to Nesse, et al.^19^ (2008).

### Outcomes

Our primary outcome was the reduction in PD (in mm) after 90 days in full-mouth parameters. Besides the full-mouth clinical parameters, the pocket stratification parameters were also assessed and divided into the number of sites with moderate (5 to 6 mm) and deep (≥7 mm) pockets per patient, PD, and CAL, allowing us to assess the Δ of reduction or gain.

Secondary outcomes were attachment gain, BoP, PI, pocket stratification assessment (moderate and deep), number of sites with deep and moderate pockets, PISA, and immunological analysis.

### Sample size

The sample size was calculated for the primary variable and change in the PD of the full mouth.^8^ Considering an ± of 5% and a type ² error of 10% (90% power) to detect a difference of at least 1 mm in PD reduction of pockets greater than 5 mm between groups, for a standard deviation of 0.61, it was estimated that 12 patients would be needed in each group. To compensate for possible losses, 15 patients were included in each group

### Immunological analysis

Gingival crevicular fluid (GCF) was collected at three sites on teeth with deep pockets (≥7 mm) to analyze cytokine levels. The cytokines evaluated were interferon gamma (IFN-γ), interleukin 1 beta (IL-1β), interleukin 6 (IL-6), interleukin 8 (IL-8), interleukin 4 (IL-4), interleukin 10 (IL-10), interleukin 12p40 (IL-12p40), interleukin 13 (IL-13), and tumor necrosis factor alpha (TNF-α). In experimental sites, the supragingival biofilm was removed, followed by light irrigation, drying (water spray/ air), and relative isolation (cotton rolls or sterile gauze). The GCF was collected with sterile absorbent paper cones (# 30, Tanari – Manacapuru, AM, Brazil) that were introduced into the pockets until they met a slight resistance and then kept for 30 seconds.^20 , 21^ The cones were transferred to Eppendorf tubes containing 200 μL of phosphate-buffered saline (PBS, pH 7.0) associated with 0.05% Tween 20 and stored in a freezer at −80°C. Levels of IFN-γ, IL-10, IL-12p40, IL-1b, IL-4, IL-6, IL-8, and TNF-α were determined using specific Multiplex Kits (HCYTOMAG-60K-06 – Human Cytokine/Chemokine Panel I, 6 plex) according to the manufacturer's instructions. Analysis was performed on MAGPIX XPONENT 4.2 TEC/NCM equipment (90275040 MAGPIX System with XPONENT 4.2).

### Statistical analysis

The data obtained after each assessment were subjected to statistical analysis. Descriptive statistics consisted of calculating means and standard deviations, and inferential statistics were performed first using the Shapiro-Wilk test to verify data normality. Since the data were not normal, nonparametric tests were used. The Kruskal-Wallis test of variance, performed in the BioEstat 5.3 software (BioEstat 5.3, Belém, PA, Brazil), and the Dunn post hoc test were used for multiple comparisons, both with a significance level of 5% to assess the inter-group comparison. The chi-square test was used to assess inter-group differences regarding qualitative variables. For the intra-group comparison, the Friedman test was used with a significance level of 5%. For data with normal distribution, one-way ANOVA tests were used.

## Results

**Figure 1:**
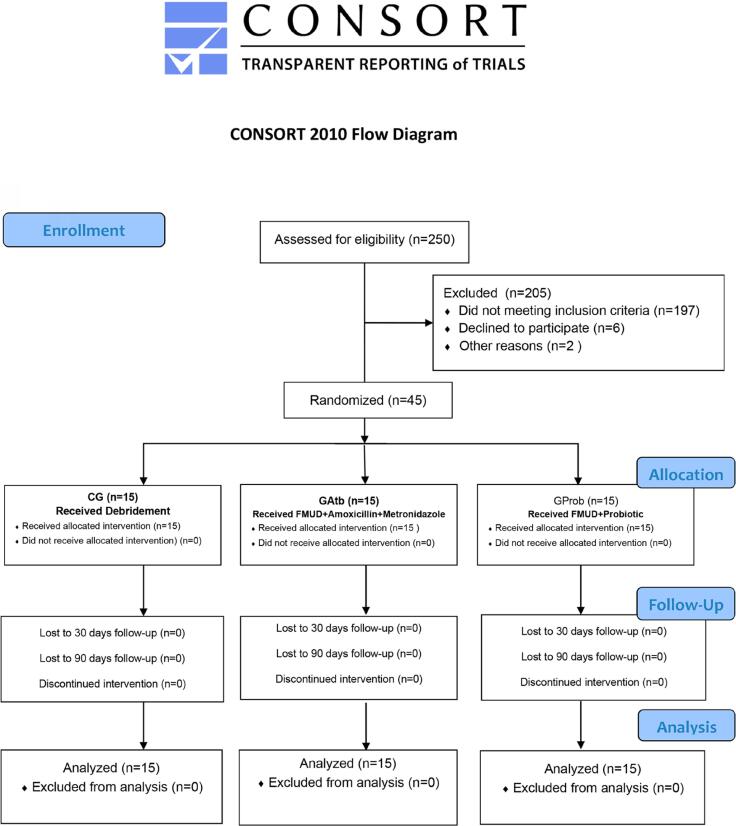
Flow chart of study

Forty-five patients were included and remained present at all follow-ups performed in the study (baseline, 30 days, and 90 days). Figure 1 shows the flowchart of the distinct phases of the study.

Table 1 shows demographic data. Groups had no statistically significant difference according to age (p=0.392) and gender (p=0.212).

**Table 1:** Demographic data of the groups

Groups	GC (n=15)	GAtb (n=15)	GProb(n=15)	p-value
Age (Years)	51.67±5.53	42.20±7.44	49.60±7.548	0.3925 a
	Male-Female	Male-Female	Male-Female	
Gender	4 11	8 7	4 11	0.212 b

(a) One-Way ANOVA test;

(b) chi-square test

### Bleeding on Probing (BoP) and Plaque Index (PI)

Table 2 shows that BoP decreased 30 and 90 days after the baseline in all intra-group comparisons. BoP reduction was more significant in the GAtb than in the GProb only at 90 days (p=0.03) of procedure. Out of the three groups, only control and probiotic groups had intra-group differences in PI. In the inter-group assessment, CG and GProb had a greater plaque index reduction (p=0.008) after 30 days. Gprob also had the best plaque control (p=0.001) after 90 days.

**Table 2:** Full-mouth clinical parameters

Variable	Period	GC(n=15)	GAtb(n=15)	GProb(n=15)	p-value (inter-group)
BoP(%)	Baseline	88.0±9.8^Aa^	93.0±10.3^Aa^	88.9±10.8^Aa^	0.36
	30 days	49.8±18.4^Ab^	35.2±11.7^Ab^	48.1±19.1^Ab^	0.08
	90 days	42.4±18.3^ABb^	28.0±6.9^Ab^	40.3±11.2^Bb^	0.03 *
PI (%)	Baseline	64.0±11.41^Aa^	70.5±19.9^Aa^	57.9±9.7^Aa^	0,1183
	30 days	46.0±7.0^Ab^	55.5±8.5^Ba^	42.8±13.1^Ab^	0.0087 *
	90 days	51.5±5.55^ABb^	54.3±7.7^Aa^	42.6±11.4^Bb^	0.0014 *
PD(mm)	Baseline	3.76±0.46^Aa^	3.66±0.42^Aa^	3.86 ± 0.69^Aa^	0.57
	30 days	3.15±0.28^Ab^	2.91±0.42^Ab^	3.27±0.51^Ab^	0.17
	90 days	3.03±0.27^Ab^	2.79±0.50^Ab^	3.13±0.45^Ab^	0.2
	Reduction(Δ)	0.73±0.30	0.87±0.46	0.73±0.38	0.43
CAL(mm)	Baseline	4.13±0.54^Aa^	4.31±1.04^Aa^	4.13±0.69^Aa^	0.94
	30 days	3.73±0.59^Aab^	3.73±0.86^Ab^	3.58±0.44^Ab^	0.75
	90 days	3.50±0.38^Ab^	3.70±0.94^Ab^	3.48±0.46^Ab^	0.98
	Gain(Δ)	0.63±0.24	0.61±0.40	0.65±0.37	0.91
GR(mm)	Baseline	0.37±0.24^Aa^	0.65±0.68^Aa^	0.28±0.20^Aa^	0.18
	30 days	0.58±0.44^ABb^	0.84±0.75^Ab^	0.34±0.16^Bab^	0.03 *
	90 days	0.47±0.26^Aab^	0.91±0.68^Ab^	0.35±0.15^Bb^	0.006 *

Kruskal-Wallis/Dunn test, p<0.05. Uppercase letters horizontally indicate Inter-group statistically significant differences; Friedman test, Lowercase letters vertically indicate intra-group statistically significant diferences;

(*) statistically significant difference.

### Probing depth (PD)

Intra-group comparisons between baseline vs 30 days and baseline vs 90 days showed a significant reduction in total PD in the 3 groups (p<0.0001). The groups had no significant difference between each other during the experimental period ( Table 2 ).

### Clinical attachment level (CAL)

In all groups, a significant full-mouth attachment gain was observed between the baseline vs 30 days and baseline vs 90 days periods (p<0.05). The groups had no significant difference between each other regarding CAL.

### Gingival recession (GR)

GR significantly increased in all groups. In inter-group comparisons, the GProb gingival recession was lower than GAtb at 30 days (p=0.03) and lower than both therapies at 90 days (p=0.006).

### Periodontal Inflamed Surface Area (PISA) Index

The PISA Index showed no statistically significant difference in the inter-group comparison; the intra-group comparison, however, showed PISA differences between baseline vs 30 days and baseline vs 90 days (p<0.05) ( Table 3 ).

**Table 3:** PISA Index data on experimental groups in the evaluated periods

Period	GC(n=15)	GAtb(n=15)	GProb(n=15)	p-value (inter-group)
Baseline	1194±209.2^Aa^	1338±433.50^Aa^	1356.52±442.1^Aa^	0.47
30 days	559.5±209.4^Ab^	440.7±180.62^Ab^	685.4±414.6^Ab^	0.18
90 days	442.7±289.7^Ab^	353.3±147.4^Ab^	510.9±217.35^Ab^	0.15

Kruskal-Wallis/Dunn test, p<0.05. Uppercase letters horizontally indicate Inter-group statistically significant differences; Friedman test, Lowercase letters vertically indicate intra-group statistically significant diferences; (*) statistically significant difference.

### Pockets stratification

#### Moderate and deep pockets

All treatments showed a significant reduction for CAL, PD, and the number of sites with moderate and deep pockets in the intra-group comparison. In the inter-group comparison, the mean probing depth and the number of sites of moderate pockets (5 and 6 mm) had no significant differences ( Table 4 ). The Δ of reduction in the number of sites also had no significant inter-group difference. Deep pockets (≥ 7 mm) had a significant difference in PD reduction, particularly in the GAtb at 90 days of procedure (p=0.02). Groups showed a significant difference in the Δ of probing depth (p=0.03), which greatly reduced in the GAtb. No significant difference was observed for CAL inter-group. The antibiotic group had less sites with deep pockets (p=0.04) after 30 days, representing a statistically significant difference. The inter-group comparison showed no statistically significant difference in Δ of PD reduction.

**Table 4:** Pockets stratification into moderate and deep

Variables	Period	GC(n=15)	GAtb(n=15)	GProb(n=15)	p-value (inter-group)
# of sites with moderate pockets (5 to 6 mm) per patient	Baseline	22.07±7.35^Aa^	20.07±11.74^Aa^	27.73±17.53^Aa^	0.27
	30 days	9.47±5.88^Ab^	8.60±6.23^Ab^	15.87±14.99^Ab^	0.17
	90 days	7.20±5.87^Ab^	6.53±5.67^Ab^	13.73±14.31^Ab^	0.23
	Reduction (Δ)	16.13±8.48	13.53±9.59	14.00±8.09	0.45
PD of moderate pockets	Baseline	5.42±0.13^Aa^	5.51±0.14^Aa^	5.43±0.19^Aa^	0.21
	30 days	4.12±0.35^Ab^	3.93±0.67^Ab^	4.12±0.45^Ab^	0.35
	90 days	3.89±0.36^Ab^	3.66±0.64^Ab^	3.92±0.49^Ab^	0.36
	Reduction (Δ)	1.54±0.41	1.85±0.65	1.52±0.43	0.21
CAL of moderate pockets	Baseline	5.43±0.10^Aa^	5.44±0.11^Aa^	5.41±0.17^Aa^	0.9
	30 days	4.56±0.46^Ab^	4.42±0.66^Ab^	4.27±0.40^Ab^	0.35
	90 days	4.25±0.34^Ab^	4.35±0.61^Ab^	4.12±0.43^Ab^	0.39
	CAL gain (Δ)	1.18±0.35	1.29±0.37	1.09±0.58	0.32
# of sites with deep pockets (≥7mm) per patient	Baseline	6.60± 4.32^Aa^	6.93±6.79^Aa^	9.90±11.35^Aa^	0.94
	30 days	1.80±1.90^Ab^	0.93±1.28^Ab^	3.07±3.95^ABb^	0.04 *
	90 days	1.93±2.12^Ab^	0.93±1.58^Ab^	1.27±1.53^Ac^	0.41
	Reduction (Δ)	4.67±3.60	6.00±6.12	8.47±10.00	0.74
PD of deep pockets	Baseline	7.90±0.76^Aa^	8.03±0.62^Aa^	7.75±0.55^Aa^	0.36
	30 days	5.28±1.02^Ab^	4.85±1.47^Ab^	5.72±0.56^Ab^	0.0522
	90 days	4.87±1.23^Ab^	4.31±0.84^Bb^	5.21±0.67^Cc^	0.03 *
	Reduction(Δ)	3.03±1.25	3.72±1.21	2.54±0.87	0.03 *
CAL of deep pockets	Baseline	8.09±0.67^Aa^	8.24±0.89^Aa^	7.75±0.69^Aa^	0.57
	30 days	5.96±1.10^Ab^	5.67±1.28^Ab^	5.89±1.10^Ab^	0.79
	90 days	5.26±1.17^Ab^	5.40±1.26^Ab^	5.50±1.12^Ab^	0.81
	CAL gain (Δ)	2.83±0.96	1.92±1.41	2.25±1.27	0.35

Kruskal-Wallis/Dunn test, p<0.05. Uppercase letters horizontally indicate Inter-group statistically significant differences; Friedman test, Lowercase letters vertically indicate intra-group statistically significant diferences;

(*) statistically significant difference;

(#) number of site.

### Adverse effects

No patient from the GProb reported any of the adverse effects described, whereas patients from the GAtb reported headache, metallic taste, nausea or vomiting, and abdominal pain. The percentage of individuals who suffered adverse symptoms was calculated from the symptoms most commonly observed in the use of the antibiotic combination ( Table 5 ).

**Table 5:** Occurrence of adverse effects during the experimental period in different groups

Adverse effect	% of subjects		
	GC (n=15)	GAtb (n=15)	GProb (n=15)
Headache	0	60%	0
Stomachache	0	7%	0
Nausea or Vomiting	0	27%	0
Metallic taste in the mouth	0	33%	0
Diarrhea or abdominal pain	0	27%	0
			
Sleepiness	0	20%	0
Itchy skin	0	0%	0
Skin wounds	0	0%	0

### Immunological parameters

**Figure 2:**
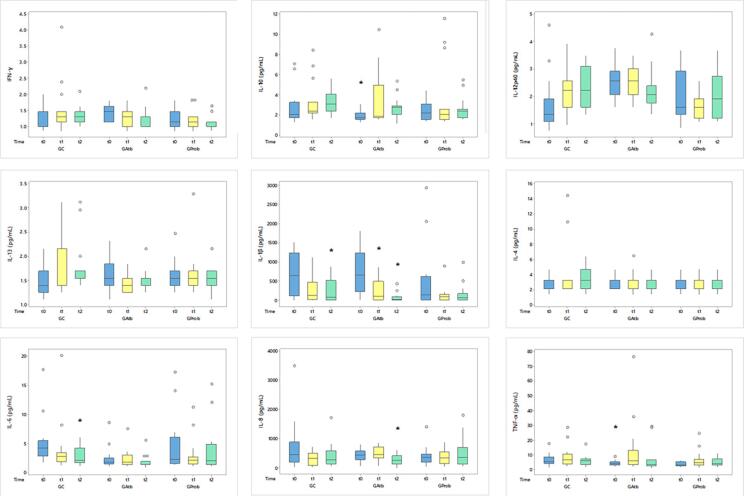
Cytokines Boxplot Chart Concentration levels of cytokines in gingival crevicular fluid. Intra-group statistically significant differences (*) Friedman test; P<0.05. Baseline (t0); 30 days (t1); 90 days (t2)

The inter-group comparison of cytokines IFN-γ, IL-10, IL-12p40, IL-13, IL-1β, IL-4, IL-6, IL-8, and TNF-α showed no differences. In the intra-group comparison, IL-1β significantly decreased in the CG after 90 days (p=0.0052) and in the GAtb at 30 and 90 days of procedure (p=0.0052). TNF-α concentration increased in the GAtb within 30 days. IL-6 significantly reduced in the CG at 90 days (p=0.0193). Within 90 days, GAtb decreased in IL-8 (p=0.014) and increased in the concentration of IL-10 ( Figure 2 ).

## Discussion

Today's society makes heavy use of medications. The literature has sought to decrease the use of antibiotics because of increasing reports of bacterial resistance associated with unpleasant adverse effects. Patients who would most benefit from probiotic therapy are those with a high plaque index and gingival inflammation. Alternative adjuvant therapies are a great option since they can improve results of periodontal therapies using different mechanisms of action and without causing adverse effects. This study was supported by previous studies^7 , 12 , 22 , 23^ on the effects of probiotics as an adjunctive method to nonsurgical periodontal therapy on clinical and immunological parameters.

According to the recently published guideline for treatment of stage I-III periodontitis^24^ , interventions such as systemic antibiotics can be included during the second step (cause-related therapy) of periodontal treatment as adjunct to subgingival instrumentations. Patients who would most benefit from the adjunct use of specific systemic antibiotics are young adults with generalized periodontitis Stage III. On the other hand, the adjunct use of probiotics was not recommended because of no statistically significant effect and limited clinical relevance (difference <0.5 mm) in PD reduction. In this study, adjuvant therapies showed no additional benefit for subgingival instrumentation after three months. However, the probiotics group reported no adverse effects, corroborating to the conclusions of Sanz, et al.^24^ (2020) that probiotics seem to be safe.

In the full-mouth assessment, the primary variable PD reduction decreased for the 45 patients included in the study, with a statistically significant intra-group difference between baseline vs 30 days and 90 days (p<0.0001) and no statistical inter-group difference, although the GAtb showed a Δ of higher reduction than other groups. The GProb had no statistical difference in reduction values, corroborating with studies that used probiotics^2^ but contradicting those that also used strains of *L. reuteri.* ^8 , 12^ The GAtb showed a reduction in PD and significant variation in deep pockets (p=0.03), corroborating with studies^4 , 23 , 25^ which found better results in deep pockets using antibiotic therapy.

The secondary variables of the study were divided into: full-mouth clinical parameters, PISA Index, pocket stratification (moderate and deep), adverse effects, and immunological parameters. These parameters allow monitoring the evolution or regression of the disease and the subgroups of moderate and deep pockets. We used different types of analysis, including PISA and immunological analysis, to establish a relationship between the inflamed sites and the cytokines involved in the treatment and to analyze adverse effects that can help verify the risk vs benefit of the therapies used. Our study was the first to compare probiotic and antibiotic therapies that used the PISA Index as an evaluation parameter. This method was proposed as a way of three-dimensionally quantifying the inflamed surface area of sites with periodontal disease, allowing us to assess the data on the disease and the resolution from after treatment. However, we noticed no differences in the analyzed therapies except for a reduction in the inflamed area for all groups during follow-up.

The use of antibiotics is known to provide better clinical parameters when the biofilm is properly removed.^22 , 26^ In this study, the antibiotic group had lower BoP values at 90 days than other groups, with a statistically significant difference (p=0.03) and a Δ of higher reduction. Despite having higher PI values than GProb and CG in the same period, the GAtb also showed a lower degree of inflammation, according to PISA values at 90 days; decreased concentrations of IL-8 and IL-1β in GCF, a pro-inflammatory cytokine released by macrophages after an infection or tissue injury;^27^ and increased concentration of the anti-inflammatory cytokine IL-10. The GProb had greater plaque index control at 30 and 90 days than the GAtb, corroborating with previous studies^8 , 12^ regarding a possible action to control the formation of plaque.

Sanz, et al.^24^ (2021) reports that subgingival instrumentation is still considered as the gold standard for the treatment of periodontitis regardless of its degree of extension and severity. It may or may not be associated with adjunct therapies during the cause-related therapy phase (second step). Clinical attachment levels showed no statistically significant inter-group difference for the association of antibiotics used in the study. Results of the probiotic group corroborate with those of previous studies,^5 , 28^ showing that therapies had attachment gain but no statistical differences between each other.

CG and the GAtb had decreased concentrations of pro-inflammatory cytokines such as IL-1β, mainly responsible for bone resorption and disease severity, 30 and 90 days after the baseline. This shows that debridement alone can effectively decrease IL-1β levels.^29 , 30^ After the therapies, the GAtb increased in anti-inflammatory cytokine IL-10, which inhibits macrophage antigens and the activation of Osteoprotegerin (OPG). This is expected after a periodontal treatment.^28^ IL-8, the cytokine which increases the differentiation of osteoclasts and attraction of polymorphonuclear neutrophils to the inflammation sites, decreased in the GAtb after 90 days.

Overall, the concentrations of pro-inflammatory cytokines INF-γ, IL-1β, IL-12p40, and IL-8 decreased whereas those of IL-10 increased. The literature reports on these changing cytokine concentration values that show no or few changes and suggests to cautiously consider the association between disease severity and cytokine levels.^29^

The GAtb showed a higher frequency of adverse effects during the medication administration period than the GProb, whose patients reported no adverse effects. The most reported adverse effects by GAtb patients were headaches (60%), metallic taste in the mouth (33%), and nausea or vomiting and diarrhea or abdominal pain (27%). The occurrence of these effects corroborates with the data in the literature.^2 , 23^

Although its effects are less evident than those of other therapies, treatment with probiotics has shown better results than debridement alone and causes no adverse effects. Therapy with antibiotics is still the most effective, despite causing adverse effects in many patients. We suggest that future studies conduct microbiological evaluations since they allowed us to understand reasons of less biofilm in our results.

This study's limitation is the reduced follow-up time since activities were suspended because of the COVID-19 pandemic. We suggest non-inferiority trials to observe if probiotics are inferior or not to antibiotics regarding benefits to the periodontal treatment and having less adverse or side effects.

## Conclusion

We conclude that none of the adjuvant therapies promoted additional benefits regarding probing depth reduction for the subgingival instrumentation after three months.
